# Influenza Epidemics in the United States, France, and Australia, 1972–1997^1^

**DOI:** 10.3201/eid1001.020705

**Published:** 2004-01

**Authors:** Cécile Viboud, Pierre-Yves Boëlle, Khashayar Pakdaman, Fabrice Carrat, Alain-Jacques Valleron, Antoine Flahault

**Affiliations:** * Institute National de la Santé et de la Recherche Médicale, Paris, France; † World Health Organization Collaborating Center for Electronic Diseases Surveillance, Paris, France; ‡ Hôpitaux de Paris, CHU Saint-Antoine, Paris, France

**Keywords:** influenza/epidemiology/mortality, pneumonia/epidemiology/mortality, comparative study, seasons, United States/epidemiology, Australia/epidemiology, France/epidemiology, statistical models

## Abstract

Influenza epidemics occur once a year during the winter in temperate areas. Little is known about the similarities between epidemics at different locations. We have analyzed pneumonia and influenza deaths from 1972 to 1997 in the United States, France, and Australia to examine the correlation over space and time between the three countries. We found a high correlation in both areas between France and the United States (correlation in impact, Spearman’s ρ = 0.76, p < 0.001, and test for synchrony in timing of epidemics, p < 0.001). We did not find a similar correlation between the United States and Australia or between France and Australia, when considering a systematic half-year lead or delay of influenza epidemics in Australia as compared with those in the United States or France. These results support a high correlation at the hemisphere level and suggest that the global interhemispheric circulation of epidemics follows an irregular pathway with recurrent changes in the leading hemisphere.

Influenza epidemics occur each year during the winter in temperate areas of the Northern and the Southern Hemispheres and result in substantial disease, death, and expense. The disease is responsible for 50 million illnesses and up to 47,200 deaths in the United States each year (*1**–**3*)*,* with similar figures in Europe (*4*–*6*). In the last century, three global epidemics, also called pandemics, occurred in 1918-19, 1957-58, and 1968-69, and recent reports estimate that the 1918-19 influenza pandemic alone may have caused as many as 50 million deaths (*7*). Changing strains of the virus are responsible for these epidemics, but little is known about what triggers the epidemic in a particular location (*2*,*8*),and how epidemics observed at different locations may be linked (*9*–*12*). Travel has been thought to be a possible cause after mathematical models based on population movements successfully explained the 1968-69 pandemic (*10*) and represented the paths of the epidemic within a country (*9*,*11*,*12*). While the cause of the geographic spread of influenza is still debated, the reasons for its seasonality are even more unclear. Although markedly seasonal, the exact timing of the winter epidemics varies from year to year. Furthermore, the interannual impact of influenza epidemics varies substantially (*1*,*3*,*4*).

Documenting patterns in the space and time dynamics of influenza epidemics is the first step in understanding the underlying mechanisms driving epidemic fluctuations over time and space (*13*). Correlations over time and space have been estimated for several animal and insect populations (*14*–*16*) and diseases such as measles (*17*–*19*) but not for influenza, despite the increasing availability of time series with the recent development of surveillance networks (*3*,*20*–*28*). We provide data and a statistical analysis for the correlations over time and space for influenza-related deaths in the United States, France, and Australia during a 26-year period which spans 1972–1997. Our goal was to determine whether influenza epidemics are correlated in impact and synchronized in time 1) at the hemispheric level and 2) at the interhemispheric level with a systematic half-year lead of one hemisphere to the other.

## Datasets and Models

### Datasets

In each country, the weekly number of deaths from pneumonia and influenza from 1972 to 1997 were computed from death certificates collected by national agencies for vital statistics (United States, National Center for Health Statistics; Australia, Australian Bureau for Statistics; France, Institut National de la Santé et de la Recherche Médicale, Service Commun 8). We used codes 470–474 and 480–486 from the International Classification of Diseases (ICD) 8th revision, before 1979, and codes 480-487 from ICD-9 from 1979 onwards to select deaths due to pneumonia and influenza. Annual population estimates were obtained from the same offices over the study period. The population sizes in 1997 in the United States, France, and Australia were 272.7 million, 58.6 million, and 18.9 million, respectively.

### Data Analysis

From the pneumonia and influenza death series, we investigated two aspects of influenza epidemics: the correlation in impact as measured by seasonal excess death rates and the synchrony in timing of the epidemic peaks.

#### Definition of Epidemic and Excess Deaths

Influenza epidemic activity is typically observed during November–March in the United States and France and June–September in Australia (*8*). We conducted analyses by using the influenza year, which runs from the first week of August to the last week of July of the next year in the United States and France and contains one annual epidemic. We considered the regular calendar year as the influenza year in Australia. From the national weekly pneumonia and influenza death time series, we defined the epidemic periods by a similar procedure to that described by Serfling and used by the Centers for Disease Control and Prevention (CDC) (*29*,*30*). In summary, we excluded 25% of the weeks in which the observed death rates were the largest to exclude epidemic periods. We then fit a seasonal regression model to the truncated series to estimate the expected baseline number of deaths in the absence of epidemic activity. A nonepidemic threshold was defined by the upper limit of the 95% confidence interval derived from the seasonal regression model. Only influenza activities that remained above the threshold for >2 consecutive weeks were included in the analysis. For each influenza year, we measured the excess deaths by subtracting the expected baseline deaths, calculated from the seasonal regression model, from the observed deaths.

#### Estimating Correlation in Excess Deaths from Influenza Epidemics

We calculated pair-wise Spearman correlation coefficients of the excess deaths estimated for each of the 26 influenza years in the study period for the three countries. For France and the United States, epidemics were paired for contemporaneous influenza years. To test for correlation between the United States and Australia or between France and Australia, we investigated two pairing scenarios: 1) influenza epidemics in Australia were systematically 6 months in advance of those of the United States and France for the 26 years considered, and 2) influenza epidemics in Australia were systematically 6 months behind.

#### Estimating Synchrony in Timing of Peaks of Influenza Epidemics

For each influenza year and each country, we defined the week of the year when the peak of the epidemic occurred as the week where the maximal number of pneumonia and influenza deaths was observed. Then, we determined the distribution of the time lags between the weeks of the peak in France and in the United States in contemporaneous influenza years, with negative values of the time lag indicating that the epidemic in France preceded that in the United States. The same distribution was calculated for Australia and the United States and for Australia and France, with the two scenarios detailed in the previous section for pairing. We expected the time lags of the peaks in the United States and France to be distributed around zero if the assumption of synchronism of the peaks held true, with a small variance indicating high synchrony. To test whether the reported distribution of the time lags was indicative of synchrony, we simulated the distribution of these lags when there was no particular synchronization mechanism between these two countries except for the seasonality of the disease.

We randomly permuted the week when the pneumonia and influenza death time series peaked in the United States for the 26 influenza years and computed the distribution of the lags with the original series from France. A p value for the existence of synchrony in the peaks was derived from this randomization procedure.

With regards to Australia, because of the change of hemisphere, we expected the week of the peak to lag by 6 months on average with that of the United States or France, regardless of the pairing scenario. Here again, the small variance of distribution of the time lags would indicate synchronism. For both pairing scenarios, we performed a randomization procedure similar to that described for comparing France and the United States to derive a p value for synchrony in the peaks.

## Results

**Figure 1:**
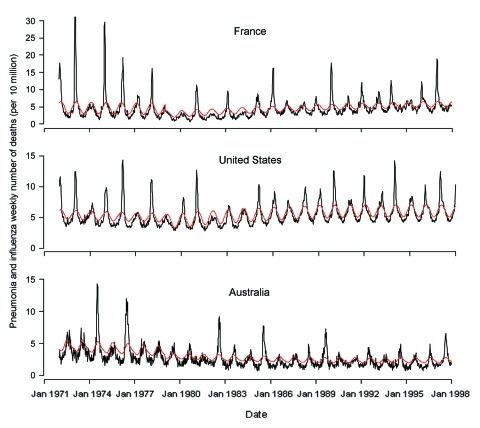
Weekly number of influenza and pneumonia deaths per 100,000 population from January 1972 to December 1997 in the United States, France, and Australia (black line). The red line represents the epidemic threshold defined by a seasonal regression.

Figure 1 depicts the raw time series of weekly number of deaths from pneumonia and influenza in the United States, France, and Australia, normalized by population size. The series show a typical seasonal pattern, with large interannual variability in the amplitude of epidemics in all three countries. The pneumonia and influenza death series for Australia appears less smooth than for the United States or France, probably because of noise effects caused by the smaller population size of Australia, representing 7% of the population size of the United States and 32% of that of France.

### Excess Deaths

For the 26 influenza years (1972–1997), we estimated a median of 2.6/100,000 population pneumonia and influenza excess deaths in the United States (range 0-6.3), 3.93/100,000 pneumonia and influenza excess deaths in France (range 0-18.3), and 1.3/100,000 pneumonia and influenza excess deaths in Australia (range 0-7.5). The rates of excess deaths were different in the three countries (Kruskal-Wallis test, p = 0.02). The Table gives the detailed annual estimates of the number of excess deaths for each influenza year. Two of 26 influenza years in the United States did not result in substantial excess pneumonia and influenza deaths, 3 in France, and 1 in Australia. The duration of the epidemic periods was similar in France and in the United States, with an average duration of 11.8 weeks in the United States (standard deviation (SD) = 0.8) and 11.4 weeks in France (SD = 0.8, p = 0.80, Wilcoxon rank test). The epidemic periods in Australia were shorter than those in the United States and in France, with an average duration of 9.6 weeks (SD = 1.0; Kruskall-Wallis test, p < 0.001).

**Table:** Number of pneumonia and influenza excess deaths and duration of influenza epidemics for each influenza year in the United States, France, and Australia, 1972–1997

Influenza season (Northern Hemisphere) ^a^	U.S. excess deaths (wk)^b^	France excess deaths (wk)^c^	Influenza season (Southern Hemisphere) ^d^	Australian excess deaths (wk)^e^
1971-72	8,100 (9)	4,200 (13)	1972	200 (11)
1972-73	11,000 (13)	9,500 (13)	1973	250 (10)
1973-74	4,100 (15)	0 (0)	1974	150 (6)
1974-75	9,000 (13)	7,000 (16)	1975	900 (14)
1975-76	13,600 (13)	4,800 (12)	1976	0 (0)
1976-77	600 (3)	1,400 (9)	1977	1,000 (16)
1977-78	10,000 (13)	2,500 (8)	1978	200 (8)
1978-79	0 (0)	300 (4)	1979	100 (7)
1979-80	4,200 (9)	0 (0)	1980	250 (13)
1980-81	8,600 (10)	3,100 (11)	1981	200 (9)
1981-82	0 (0)	0 (0)	1982	0 (0)
1982-83	1,400 (5)	1,800 (10)	1983	700 (15)
1983-84	700 (4)	0 (0)	1984	250 (12)
1984-85	7,000 (11)	2,300 (15)	1985	150 (8)
1985-86	6,000 (13)	4,700 (18)	1986	650 (15)
1986-87	2,000 (7)	900 (9)	1987	0 (0)
1987-88	8,700 (18)	700 (8)	1988	100 (7)
1988-89	7,000 (16)	1,000 (9)	1989	200 (9)
1989-90	10,400 (14)	4,300 (11)	1990	750 (17)
1990-91	3,300 (12)	1,000 (10)	1991	100 (6)
1991-92	6,800 (10)	2,900 (16)	1992	50 (3)
1992-93	6,300 (16)	2,100 (16)	1993	300 (14)
1993-94	9,800 (11)	2,600 (11)	1994	50 (3)
1994-95	5,300 (15)	900 (10)	1995	300 (10)
1995-96	6,300 (16)	2,500 (17)	1996	100 (5)
1996-97	11,400 (18)	4,500 (13)	1997	250 (10)
^a^Excess deaths reported from August of a given year to July of the next year during an influenza epidemic (e.g., for row labeled 1971-72, excess deaths occurring from August 1971 to July 1972). ^b^Population, 272.7 million. ^c^Population, 58.6 million. ^d^Excess deaths reported from January to December of a given year during an influenza epidemic (e.g., for row labeled 1972, excess deaths occurring from January 1972 to December 1972). ^e^Population, 18.9 million.				

### Correlation in Excess Deaths

**Figure 2:**
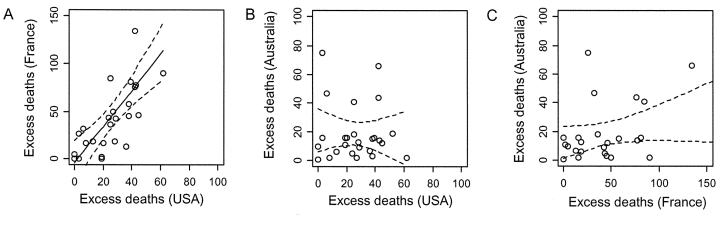
Correlation in the impact of influenza epidemics for 26 influenza years (1972–1997), measured by the annual number of pneumonia and influenza excess deaths. A, excess deaths per million in France (y axis) and the United States (x axis) in contemporaneous winters: Spearman correlation coefficient = 0.76 (p < 0.001). C, Excess deaths per million in Australia (y axis) and the United States (x axis), considering the scenario in which the influenza season in Australia is systematically 6 months before that of the United States: Spearman correlation coefficient = 0.14 (p = 0.50). C, excess deaths per million in Australia (y axis) and France (x axis), considering the scenario where the influenza season in Australia is systematically 6 months before that of France: Spearman correlation coefficient = 0.37 (p = 0.06). We obtained similar results as in B and C, when we considered a reverse scenario in which the influenza season in Australia was systematically lagging 6 months behind that in the United States or France.

The correlation coefficient for excess deaths in the United States and France in contemporaneous influenza years was high (Spearman’s ρ = 0.76 for the United States and France, N = 26, p < 0.001; Figure 2a). On the contrary, excess deaths in Australia were not correlated with excess deaths in France or the United States in the scenario in which Australia was systematically 6 months in advance (Spearman’s ρ = 0.14 for Australia and the United States, p = 0.50 and ρ = 0.37 for Australia and France, p = 0.06; Figure 2b and c) or in the scenario in which Australia was systematically 6 months behind (Spearman’s ρ = 0.15 for Australia and the United States, p = 0.47 and Spearman’s ρ = 0.17 for Australia and France, p = 0.37).

#### H3 Synchrony in Timing of Peaks

**Figure 3:**
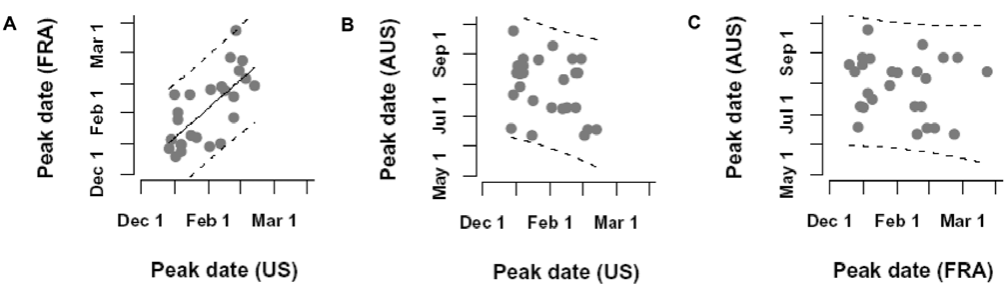
Synchrony in the timing of the peaks of influenza epidemics for 26 influenza years (1972–1997). Correlation between the week of year of the epidemic peak A) in the United States (x axis) and in France (y axis). B, in the United States (x axis) and in Australia (y axis). C, in France (x axis) and in Australia (y axis). Panels b) and c) illustrate the scenario in which the influenza season in Australia is systematically 6 months before that of the United States or France. Similar results are obtained for the reverse scenario.

**Figure 4:**
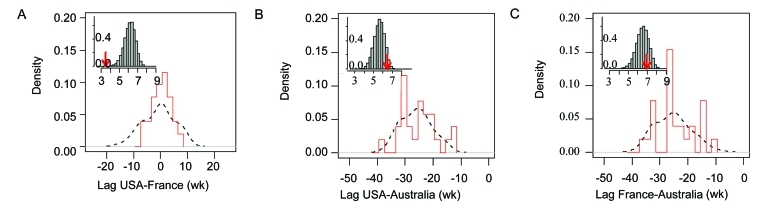
Synchrony in the timing of the peaks of influenza epidemics for 26 influenza years (1972–1997). Distribution of the time lags between the epidemic peaks in weeks (main plot). The red bars represent the observed lags, and the dashed line represents the distribution of lags obtained by permutations. (inset plot) Distribution of the standard deviation of permuted lags under the assumption of no synchrony. Red arrow indicates the standard deviation in the observed data. A, United States and France, b) United States and Australia, C, France and Australia. Panels B and C illustrate the scenario in which the influenza season in Australia is systematically 6 months before that of the United States or France. Similar results are obtained for the reverse scenario.

In France and in the United States, in the 26 influenza years, the median time lag between the weeks when the peak occurred was half a week (N = 26, range –8 through 6) reflecting a high level of synchrony (Figure 3a). The peak occurred earlier in the United States in 11 of the 26 epidemic seasons, earlier in France in 13 epidemic seasons, and on the same week for both countries in 2 epidemic seasons. Influenza epidemics did not peak earlier in France more often than in the United States, or vice versa (chi square, p = 0.66). Of the 100,000 randomizations we performed by permuting the week of the peak, 2 gave a variance for the time lags lower than that reported for the original data (Figure 4a; p < 0.001). Therefore, these results show synchronism in the times of peaks between France and the United States.

In the scenario in which the influenza season in Australia was systematically 6 months in advance of that in the Northern Hemisphere, the median time lag between the peaks in Australia and in the United States was 27 weeks (range 14-39) and 26 weeks between those in Australia and in France (range 15-41; Figure 3b and c). In the scenario in which the influenza season in Australia was systematically 6 months behind that in the Northern Hemisphere, these lags were 25.5 weeks (range 13-36) and 24.5 weeks (range 13-34), respectively. The simulated distributions obtained from the 100,000 permutations of the week were not different from the observed distributions (in the scenario in which the influenza season in Australia was 6 months behind, p = 0.70 and p = 0.79 for the United States and France, respectively [Figure 4b and c] and in the scenario in which it was 6 months in advance, p = 0.13 and p = 0.12). For these two pairing scenarios, no synchrony in the peaks was evident.

## Discussion

The results of this study favor a high level of correlation in amplitude and synchrony in the timing of influenza epidemics in France and the United States. No correlation or synchrony was found between Australia and the United States, or between Australia and France in the two scenarios in which Australia systematically led or lagged behind the global interhemispheric circulation of epidemics by 6 months.

The level of correlation in amplitude evidenced here depends on the statistical method used for estimating seasonal excess deaths. We have used a similar procedure to that described by Serfling and used by the CDC for estimating excess deaths (*3*,*29*). In our study, periods of increased influenza activity were excluded from the baseline model by discarding upper marginal values that were above a somewhat arbitrary cutoff (25th percentile of the distribution), and we found a high correlation between excess deaths in the United States and France (Spearman’s correlation coefficient ρ = 0.76). The correlation remained stable with a lower cutoff set at 10%: in this analysis the correlation coefficient was also 0.76. Moreover, our excess death estimates are well in line with those published for the United States (*3*,*31*,*32*) and for France by using a different statistical approach (*4*). For the United States and France, the correlation coefficients between our estimates of pneumonia and influenza excess deaths and the most recently published estimates were 0.87 and 0.94, respectively, with similar orders of magnitude (*3*,*4*). No comparable estimates were available for Australia. Pneumonia and influenza death series have been used traditionally since the work of William Farr in the late 1840s to quantify the effect of influenza epidemics in terms of death (*33*) because severe complications are triggered by influenza infection and result in death, in particular bacterial pneumonia (*34*). The use of pneumonia death series might introduce additional background noise in estimating excess deaths caused by influenza, especially because of the high level of activity in the summer and the upward trend observed since the early 1980s (*6*). We performed a sensitivity analysis by restricting our calculations of excess deaths to deaths coded as influenza only and retrieved a similar correlation between the United States and France (Spearman’s ρ = 0.75). However, we do not capture the total impact of influenza by analyzing only pneumonia and influenza death, which accounts for only part of the overall deaths from influenza (*4*,*34*). Therefore, we probably cannot rely strictly on our absolute estimates of excess deaths to study the overall impact of influenza epidemics on death. However, pneumonia and influenza deaths series provide a robust and unbiased indicator of the severity of influenza epidemics for between-country comparisons. In addition, with an indicator as simple and crude as the raw pneumonia and influenza deaths observed during the week of the peak, the correlation remains of the same order (Spearman’s ρ = 0.73).

Demography may contribute to the lack of correlation and synchrony between Australia and the United States or between Australia and France. While the age structures of the populations in the three countries are similar, the population sizes are different. Discrepancies in the levels of vaccination might also play a role. However, the number of doses of influenza vaccine distributed was similar in France and the United States from 1980 until the early 1990s but doubled in recent years in the United States (*35*). The level of vaccination in Australia was about half of that of France and the United States from 1980 until 1990 when it was similar to that of France in 1997 (*35*). Therefore, no obvious link existed between trends in vaccination and our results.

An important limitation in the analysis of correlation and synchrony between countries in different hemispheres lies in the arbitrary choice for pairing epidemics. We investigated two extreme scenarios in which influenza epidemics in Australia were systematically 6 months ahead of those in France or the United States or systematically 6 months behind. We found no correlation or synchrony in either one. We cannot rule out a more complex interhemispheric pathway with recurrent changes in the leading hemisphere. We searched for an optimal theoretical pairing, allowing each epidemic in Australia in our simulations to pair with either the preceding or following epidemic in the United States (or France) to maximize the correlation in excess death. For optimal pairing, the correlation coefficients obtained were in the same order of magnitude as those observed between France and the United States (Spearman’s ρ = 0.66 for the United States and Australia and Spearman’s ρ = 0.71 for Australia and France).

Few reports document the circulation of influenza virus on a worldwide scale. Hope-Simpson studies of death, disease, and virologic datasets suggest that the circulation of influenza strains (and the epidemics they cause) may follow a given pathway during several years (e.g., Southern to Northern Hemisphere) and subsequently shift to the other pathway (e.g., Northern to Southern Hemisphere) (*36*). This finding agrees with the absence of significant time and space correlations found in this work between countries in different hemispheres when epidemics were paired 6 months ahead or behind. If this absence of correlation between the two hemispheres, observed in death records limited to an interpandemic period and a limited set of countries, held true in the case of a pandemic, information on previous routes of transmission derived from historical epidemics (*10*,*37*) could prove of little use for planning the route of future epidemics or pandemics on a worldwide scale.

Our study nevertheless strongly suggests that influenza epidemics are correlated in amplitude and synchronized in timing in the Northern Hemisphere, and collection and analysis of additional data is underway in other countries of Europe and North America. Prevaccination measles epidemics in different locales of the United States and the United Kingdom were also highly correlated in time and space, a situation that evolved to the observed absence of correlation in the last 2 decades after the level of vaccination increased from 50% to 90% (*17*,*19*). This lack of correlation is reportedly promoting persistence of the disease (*19*). Space and time correlations of influenza epidemics may follow the same pattern as vaccine coverage increases. How the very high rate of antigenic evolution of influenza virus, a feature not found in measles, could impact on this change requires further study, all the more as antigenic variations explain partly the impact of the disease (*3*,*19*,*38*–*40*). The current plan to control influenza advocated by the World Health Organization promotes vaccine use and, in case of emergence of a pandemic virus, extensive use of antiviral drugs and vaccine, assuming that the vaccine could be produced in a timely fashion (*41*). The impact of an expected loss of correlations in influenza epidemics resulting from increased vaccine use should be further investigated, especially by mathematical modeling.

In conclusion, two factors have been reported to drive the space and time correlations of epidemics: population movements and environmental issues, such as climate or weather conditions (*14*–*16*). Population movements are assumed to play a key role, though not quantified, in the global spread of influenza epidemics (*8*,*10*), but the role of environmental factors and virus circulation between the Southern and Northern Hemispheres (*42*–*44*) remains to be clarified. Large-scale molecular epidemiologic studies of influenza viruses, sampled from locations in different hemispheres, that investigate the circulation pathways could assist in the understanding of the reasons for the seasonality of the disease (*44*). Such large-scale studies seem feasible in light of the recent plan for a global sentinel laboratory that could centralize genotype and archive samples collected worldwide (*45*).
